# Determinants factors to Pap smear screening among married women in a city of South Iran: applying the BASNEF model

**DOI:** 10.1186/s12905-020-01102-6

**Published:** 2020-10-20

**Authors:** Rahimeh Momeni, Zahra Hosseini, Teamur Aghamolaei, Amin Ghanbarnejad

**Affiliations:** 1 Health School, Hormozgan University of Medical Sciences, Bandar Abbas, Iran; 2 Tobacco and Health Research Center, Hormozgan University of Medical Sciences, Bandar Abbas, Iran; 3 Social Determinants in Health Promotion Research Center, Hormozgan Health Institute, Hormozgan University of Medical Sciences, Bandar Abbas, Iran

**Keywords:** Cervical cancer, Pap smear, Women, BASNEF model, Iran

## Abstract

**Background:**

Cervical cancer is known to be preventable because of the long pre-invasion period and the availability of appropriate screening methods. Pap smear is a selective screening approach, which is not taken seriously enough by many women.

**Methods:**

This cross-sectional, descriptive, analytical study was performed using electronic health records of 202 women visiting the health centers chosen through the systematic sampling method. The data collection tool contained items on demographic information, awareness regarding cervical cancer, and the beliefs, attitudes, subjective norms, and enabling factors (BASNEF) model constructs. Data were analyzed using the linear regression analysis, logistic regression, and multivariate regression analysis with backward selection in SPSS, version 18.

**Results:**

Based on the results, more than half of the women had never had a Pap smear test. Of the 202 women, only 14.8% had repeated the Pap smear test at the standard interval. Attitudes and subjective norms predicted the intention to have a Pap smear test among the eligible women. Overall, 10% of the changes in behavioral intention were explained by attitudes and subjective norms. In the BASNEF model, the behavioral intention was one of the most important factors that affected compliance with the Pap smear test among the eligible women.

**Conclusion:**

Based on the results of this study, it is possible to improve screening behaviors among women through proper planning to increase awareness and improve attitudes, subjective norms, enabling factors, and behavioral intention. Pap smear plays an important role in controlling cervical cancer.

## Background

The global burden of cervical cancer is not equally distributed throughout the world, and more than 80% of new cases of this disease occur in developing countries. The rate of cervical cancer has significantly reduced in developed countries due to performing cytology-based screening programs in recent years [1]. Studies in developing countries have shown that only 4.1% of the women aged 18–69 years old had regularly undergone cervical cancer screening over the past 3 years [2].

The incidence of this cancer in Iran is lower than the global average, but due to the increased smoking, hookah and drug abuse among the younger generation, lifestyle changes and increased risky behaviors it is expected that HPV will increase and the incidence of this cancer in Iran may also increase [3].

Cervical cancer is known as a preventable malignancy because of its long pre-invasion period, access to appropriate screening programs, and effective treatments for primary lesions. The risk factors for this disease include marriage in early ages, having a sexual relationship before the age of 18, history of multiple marriages, history of multiple pregnancies, smoking, intercourse with uncircumcised men, and some sexually transmitted genital tract infections [4–6]. The most important risk factor for cervical cancer is human papillomavirus (types 16 and 18) [7].

The difference in cervical cancer mortality between developing and developed countries is directly related to the Pap smear test compliance. Thus, implementing programs to improve health-related behaviors, beliefs, and awareness regarding the Pap smear test is essential [8]. These programs can reduce the incidence of cervical cancer and its mortality up to 79% and 70%, respectively [9]. According to previous studies, early diagnosis and treatment in the pre-cancerous phase can prevent cervical cancer [10]. Thus, cytology-screening programs are very effective in the early diagnosis of this cancer [11–13].

According to a report by the Ministry of Health and Medical Education of Iran, all women should have cervical cancer screening after their marriage. Women aged 35–54 years are at a greater risk, but screening is also performed for married women aged more than 18 years old at 3 years after their marriage. After three normal and reliable samples of Pap smear and lack of any risks, the Pap smear test is repeated every 3 years [13]. According to some studies, the lack of knowledge and awareness regarding cervical cancer and the related behaviors to prevent it are the main reasons for non-compliance with the Pap smear test [9].

Cervical cancer screening programs in Iran are performed at three levels: The first level is aimed at identifying suspected or suffering from cervical cancer and providing appropriate services to them. This is done at health centers by health care providers. The second level is for people who are referred to a surgeon or hospital for any reason. At this level a more thorough evaluation is done. The level three is specialized. More complete services (Such as: blood tests, surgery and radiotherapy) are provided to these people [14].

One of the reasons for the failure of educational programs in improving healthy behaviors is the lack of attention to the factor affecting behaviors and psychosocial models as specific intellectual frameworks in educational programming. In this study, the beliefs, attitudes, subjective norms, and enabling factors (BASNEF) model was used to predict the effective factors of cervical cancer screening behavior. This model is used to study behaviors and program to change them. It also determines the effective factors in deciding to perform a behavior, the important factors that lead to healthy behaviors, and norms, social pressures, and attitudes towards a behavior. The BASNEF model is based on Fishbein’s theory regarding attitude structures, behavioral intention, subjective norms, and enabling factors such as the skill of performing a behavior, time, and expenditure [15]. The best predictor of a behavior is the individual’s intention to do it; on the other hand, attitudes and subjective norms can affect performing a behavior through their influence on behavioral intention [16]; this pattern has been specified in various studies [17, 18].

As mentioned before, screening plays an important role in the early detection of cervical cancer; however, in the city of Dayyer, Bushehr Province, Iran, the rate of women's referral for screening is very low because of social and cultural atmosphere of this city and women’s shame of having the Pap smear test. The women eligible for Pap smears in 2016 who had electronic records in Dayyer’s clinics was 4100, of whom only 80 had Pap smears. Considering the scarcity of studies on this issue, the present study was designed to predict the related factors on adopting pap-smear behavior in married women based on the BASNEF model and to identify the current status. The results of this study can help to design the necessary educational programs and counseling services, to reduce barriers to screening and improve the quality of health services for women and society.

## Methods

### Study design, population and sampling

Dayyer city have 2 health centers: Hazrat Mahdi and Emam Khomeini health centers. This cross-sectional, descriptive, analytical study was performed among all the 20 to 49-year-old women visiting the health centers of Hazrat Mahdi and Emam Khomeini in Dayyer city. The total population of women the ages of 20–49 were 4100. Accordingly, 202 women who had electronic health records were selected through the systematic random sampling method from two health centers. The sample size was calculated considering the formula of estimating the sample size in descriptive studies with the confidence interval of 95%. Two health centers of the city were included in the study and according to the covered population, the subjects were randomly selected from women with electronic health records. Samples were selected by alternate (ie first person selected, second no, third yes, fourth no, etc.) and continued until the desired sample size was reached.

The review of the electronic files took a month. From the 1st of January until the end of January 2017. The names and phone numbers of the people are in the file. After determining the number of samples, we contacted them. We explained the purpose of the study and invited them to medical centers to complete the questionnaires. Questionnaires were designed based on BASNEF model constructs were completed through interviews with women in the centers and its data analysis took about 6 months.

The inclusion criteria included the lack of disability or inability to answer the questions, at least elementary education, lack of history of hysterectomy, age over 18 years, willingness to participate, and lack of cervical cancer or other cancers. The exclusion criteria were pregnancy and unwillingness to participate in the study.

### Data collection tools

The data collection tool was a questionnaire including three parts (Additional File 1). The first part contained items on demographic information including age, marital status, education, spouse’s profile, pregnancy profile, and history of the Pap smear test. The second part consisted of 15 questions regarding Knowledge about cervical cancer and its preventative factors. The items were rated using three options (true, false, and I don’t know); correct responses were given one score while incorrect and I do not know responses were given a score of 0. The third section contained questions on the BASNEF model structures. The items that explored the BASNEF constructs (except for behavior), were rated on a 5-level Likert scale ranged from 0 to 4. The 5 choices included were totally agree, agree, undecided, disagree and totally disagree. The measurement scale for behavior, was yes and no.

Validity and reliability of one part of the questionnaire (awareness, attitudes, and behavior) have been approved in previous studies based on the Health Belief Model [19] and the other questions regarding the BASNEF model structures were designed by the researcher through studying the related papers and documents and using the suggestions of a panel of experts. The questionnaire was given to 10 faculty members of health education and gynecologists and their comments were applied to the questionnaire and 15 women that met the research population characteristics were asked to comment on the clarity, legibility, and relevance of the item. As a result, no item was omitted, but some were corrected.

Cronbach’s alpha test was used to establish the reliability of the BASNEF questionnaire and was comprised of the following components:

*Attitude* This structure was represented by 11 items, such as: “If I have cervical cancer, I prefer not to know about it”. The scores of the items were summed up and the overall scores ranged from 0 to 44. Cronbach’s alpha test for this component was estimated at 0.71.

*Subjective Norms* There were 16 items included within this component, such as: “My husband advises me to do a Pap smear test.”. The scores of the items were summed up and the overall scores ranged from 0 to 64. Cronbach’s alpha test for this component was estimated at 0.87.

*Enabling Factors* This structure was comprised by 6 items, such as: “I do not have enough time to do a Pap smear test”. The scores of the items were added up and the overall scores ranged from 0 to 24. Cronbach’s alpha test for this component was estimated at 0.78.

*Behavioral Intention* This component was represented by 5 items, such as: “in order to reduce the risk of cervical cancer, I intend to keep cleanliness my genital organ”. The scores of the items were added up so the overall scores would range from 0 to 20. Cronbach’s alpha test for this component was estimated at 0.75.

*Behavior* Behavior was measured by 1 items: “I perform the Pap smear test regularly (Once a year up to 3 years, if there is no problem every 3 years)”.

### Ethics approval

The study protocol was approved by the Ethics Committee of Hormozgan University of Medical Sciences (HUMS.REC.1396.116). Prior to performing the study, informed consent was obtained verbally. Participation in the research did not have any financial burden for the participants. The respondents were fully informed of the purpose of the study and were ensured of the confidentiality of their personal data. Participants were also free to withdraw from the study at any stage.

### Data analysis

Descriptive indices including mean and standard deviation for quantitative variables and number and percent for categorical variables were reported. Pearson correlation were used for investigate the correlation between BASNEF constructs. Then linear regression were used to assess the association between behavioral intention and other constructs. Logistic regression were performed to investigate the associated factor with behavior. All analysis were performed in SPSS version 18.

## Results

All participants were residents of Dayyer city. The mean age of the subjects was 32.75 ± 6.82 years (range: 20–49 years). Most of the participants were housewives with secondary education (Table 1). Moreover, 32.7% (66 people) of the subjects had a history of the Pap smear test, of whom 2% (4 people), 4.5% (9 people), 2.5% (5 people), 5.9% (12 people), and 17.8% (36 people) had performed the test 1 month, 3 months, 6 months, 1 year, and more than 1 year before the study, respectively. Only 30 out of the 202 subjects had performed the Pap smear test regularly. Mothers or sisters of 34.2% (69 people) of the subjects had a history of the Pap smear test.

**Table 1:** Specifications of the participants in the study

Variable	Number (%)
Age	
20–30	82 (40.5%)
31–40	92 (45.5%)
41–49	28 (13.8%)
Education	
Illiterate	1 (0.5%)
Elementary	39 (19.3%)
Guidance	46 (22.8%)
Diploma	80 (36.9%)
Associate degree	5 (2.5%)
Bachelor	30 (14.9%)
Master’s degree	1 (0.5%)
Occupation	
Housewife	177 (87.6%)
Employed in public sector	21 (10.4%)
Employed in private sector	4 (2%)

Descriptive statistics and the correlation coefficients between awareness and the BASNEF model structures are presented in Table 2. According to the results, awareness, attitudes, subjective norms, enabling factors, and behavioral intention of performing Pap smear showed a positive significant correlation (*P* < 0.01).

**Table 2:** Pearson correlation coefficient between structures of the BASNEF model

	Knowledge	Attitude	Subjective norms	Enabling factor	Behavioral intention	Mean ± SD
Knowledge	1					5.83 ± 3.34
Attitude	0.359**	1				27.92 ± 5.93
Subjective norms	0.328**	0.533**	1			42.20 ± 9.39
Enabling factor	0.459**	0.402**	0.468**	1		14.16 ± 5.64
Behavioral intention	0.173*	0.288**	0.306**	0.250 **	1	14.00 ± 3.34

**Significant at level 0.01; *Significant at level 0.05

Multivariate regression analysis could predict 10% of the variance in the intention to perform the Pap smear test based on different BASNEF model structures. In addition, the structures of attitude and subjective norms had a higher predictive power in determining behavioral intention (*P* = 0.02; Table 3).

**Table 3:** Multivariate linear regression analysis predicts the behavioral intention of the BASNEF model

Variable	Unstandardized coefficient	Standardized coefficient	*P* value	R-squared
Attitude	0.109	0.186	0.025	0.1
Subjective norms	0.067	0.185	0.025	
Constant	8.18	–		

For investigating the chance of performing a Pap smear test based on the BASNEF model structures, univariate and multivariable logistic regression was used. The variables of awareness, attitude, subjective norms, enabling factors, and behavioral intention entered into the model as independent variables and the behavior of attending the Pap smear test entered as the dependent variable. In univariate analysis, all the constructs except knowledge had significant effect on doing Pap smear.

Multivariate model could explain 32% of variation in Pap Smear based on Cox & Snell R-square provided by software output. Among the mentioned variables, the behavioral intention was the strongest predictor of compliance with the Pap smear test according to multivariate model (Table 4).

**Table 4:** Univariate and multivariate analysis for predicting the behavior of performing Pap smear by BASNEF model structures

Variable	Range of scoring	Univariate		Multivariate	
		Odds ratio (95% CI)	*P* value	Odds ratio (95% CI)	*P* value
Knowledge	0–15	1.14 (1.00–1.30)	0.05	0.92 (0.74–1.17)	0.53
Attitude	0–44	1.14 (1.06–1.23)	0.001	1.03 (0.90–1.18)	0.67
Subjective norms	0–64	1.15 (1.08–1.22)	< 0.001	1.08 (1.01–1.16)	0.032
Enabling factor	0–24	1.17 (1.08–1.26)	< 0.001	1.09 (0.95–1.25)	0.20
Behavioral intention	0–20	2.45 (1.77–3.38)	< 0.001	2.29 (1.57–3.35)	< 0.001
				R-square = 0.32	

*CI* confidence interval

## Discussion

In this study, we sought to evaluate the predictive factors of attending the Pap smear screening in married women based on the BASNEF model. The study results showed that more than half of the women under study had never attended Pap smear screening programs. In addition, the findings of Ahmadipour and Farshbaf Khalili indicated that women’s attendance in the Pap smear screening was low and more than half of the eligible women had never performed a Pap smear test [2, 13]. Mohebi et al. [20] showed in their study that a significant portion (more than one-third) of their participants had not participated in cervical cancer screening programs. It was also indicated in the Roanda’s study in 2017 that only 13.3% of asymptomatic women had attended the screening, and more than one-third of the symptomatic women had never performed a Pap smear test [21], which is consistent with our results. However, Louie and Solomon showed in their studies that 93% and 85% of women had regularly attended the Pap smear test, respectively [22, 23]. Cultural differences, technological developments, and the difference in the availability of amenities could explain this discrepancy. In Dayyer, most women did not attend the Pap smear screening because they cared about their children more than themselves, did not devote sufficient time to self-care and check-ups, felt ashamed of vaginal examination, had no access to laboratories, and could not afford the high costs of the examination [3, 24].

In Iran, women have to pay for Pap smears. Pap smears are performed at health centers by health care providers. The cost of providing the equipment needed to perform the test (such as: Speculum, Lam, Spray Fixer and Brush) as well as the cost of the lab to interpret the test specimens should be pay by women. There is also no comprehensive educational program in our country on informing about cervical cancer and Pap smear testing, lowering the rate of Pap smear can be for these reasons.

In addition, the results showed that only 14.8% of the 202 subjects in this study had repeated the test at standard intervals. The results of Mohebi et al. showed that 11.5% of the participating women had attended the Pap smear screening regularly, and 51.87% had participated in cervical cancer screening programs irregularly [20]. Moreover, 24.4% of women had performed the Pap smear test more than twice in Qayoumi et al. study [7], which is in agreement with our findings, but in Chorley et al. study, most women had participated in the Pap smear screening program at least once [25].

In this study, attitudes and subjective norms were the predictors of behavioral intention of performing Pap smear among the eligible women, and in general, they predicted 10% of changes in behavioral intention. The results of Moradi et al. [16] showed that subjective norms and attitudes were the predictors of the intention to perform the Pap smear test among the women visiting health care centers. In the study of Keshavarz et al. [26], husbands were reported as the most important sources of subjective norms. In addition, in a study by Bahri et al. [8], 87.3% of women had positive attitudes toward the Pap smear test. A study performed on Taiwanese women showed that subjective norms were the predictors of intention of cervical cancer screening [27]. In a study by Ogilivie et al. [28], attitudes and subjective norms were the predictors of intention of cervical cancer screening.

Among the variables of the BASNEF model, the behavioral intention was the most effective factor on Pap smear screening behavior and in studies conducted based on the behavioral intention model, the predictive power of behavioral intention was found affected by attitudes, subjective norms, and perceived behavioral control, in sequence [29]. The high predictive power of behavioral intention was consistent with the findings of Ouji et al. [15] who showed that among behavioral intention and enabling factors, only was behavioral intention the predictor of physical activity behavior. In addition, Baqiani Moqadam et al. showed that behavioral intention and enabling factors were directly effective on self-monitoring behavior, and the effect of behavioral intention was stronger. They ascribed that enabling factors were also effective on behavior through their indirect effects on intention [17]. In a study by Lee et al. [30], a significant correlation was observed between intention and self-management behavior.

The present study had some limitations including the self-report nature of the data. In addition, due to the cross-sectional design of the study, causal effects could not be proved in this study. Illiterate and illiterate women were included in the study. The barriers to performing Pap smears may be different in them, which may not be mentioned in the present questionnaire.

## Conclusion

Based on The results subjective norms and behavioral intention were the two predictors of behavioral intention. Cervical cancer can be prevented through identification and treatment of pre-invasive lesions and the consequences of its late diagnosis can be reduced through raising women’s awareness via implementing educational programs based on the BASNEF model, evaluating enabling factors, and eliminating barriers to the Pap smear screening.

## Supplementary information

**Additional file 1:** Pap smear questionnaire based on BASNEF model.

## Data Availability

The datasets generated and/or analysed during the current study are not publicly available, but are available from the corresponding author upon reasonable request.

## Abbreviations

BASNEF: Attitude, subjective norms, enabling factors, behavioral intention, behavior
CI: Confidence interval
