# Supplementary material for: Determinants factors to Pap smear screening among married women in a city of South Iran: applying the BASNEF model
Source: BMC Womens Health. 2020 Oct 20;20:237. doi: 10.1186/s12905-020-01102-6 (PMC7576855; doi:10.1186/s12905-020-01102-6)
Supplement: Supplementary file 1 — Additional file 1: Pap smear questionnaire based on BASNEF model. [file 12905_2020_1102_MOESM1_ESM.doc]

Knowledge

| I do not know | False | Correct | Items |  |
| --- | --- | --- | --- | --- |
|  |  |  | Cervical cancer is preventable | 1 |
|  |  |  | Abnormal bleeding is not a symptom of cervical cancer | 2 |
|  |  |  | Pap smear testing is one of the early detection methods for cervical cancer | 3 |
|  |  |  | The best time to do a Pap smear test is twelve days after the end of the mensturation | 4 |
|  |  |  | Pap smear tests can be performed at health centers. | 5 |
|  |  |  | Pap smear test is essential for women with abnormal bleeding | 6 |
|  |  |  | Pap smear test is only required for women of childbearing age (15 to 45 years) | 7 |
|  |  |  | Marriage at an early age increases the risk of cervical cancer | 8 |
|  |  |  | Early pregnancy at an early age (under 21 years) increases the risk of cervical cancer | 9 |
|  |  |  | Family history of cervical cancer increases the risk of cervical cancer | 10 |
|  |  |  | Taking contraceptive pills is associated with cervical cancer | 11 |
|  |  |  | Failure to comply with genital health increases the risk of cervical cancer. | 12 |
|  |  |  | If one of a couple has sexually transmitted disease increases the risk of cervical cancer. | 13 |
|  |  |  | Vitamin A, Vitamin C and Folic Acid Deficiency in Diet Increases Risk of Cervical Cancer | 14 |
|  |  |  | Tobacco smoking (cigarettes, hookah, etc.) is associated with cervical cancer. | 15 |

Attitude

|  | Please comment on the following questions | I agree very much | I agree | No idea | I disagree | I disagree very much |
| --- | --- | --- | --- | --- | --- | --- |
| 1 | It is not affordable to do a Pap smear |  |  |  |  |  |
| 2 | The Pap smear test is very simple |  |  |  |  |  |
| 3 | Pap smear test is easier than cervical cancer treatment. |  |  |  |  |  |
| 4 | If I go for a uterine examination, I'd rather have a Pap smear test. |  |  |  |  |  |
| 5 | I have doubts about the effect of Pap smear on early diagnosis of cervical cancer. |  |  |  |  |  |
| 6 | I'd rather have a Pap smear before cervical cancer symptoms. |  |  |  |  |  |
| 7 | Sampling is hard for me and I hate every examination. |  |  |  |  |  |
| 8 | If I have cervical cancer, I'd rather not be aware of it |  |  |  |  |  |
| 9 | Cervical cancer affects my family relationships and my job. |  |  |  |  |  |
| 10 | I only do Pap smear tests if forced |  |  |  |  |  |

Subjective Norms

|  | Please comment on the following questions | I agree very much | I agree | No idea | I disagree | I disagree very much |
| --- | --- | --- | --- | --- | --- | --- |
| 1 | My wife protests if I see a doctor for Pap smear |  |  |  |  |  |
| 2 | My sister or my mother protests if I go to the doctor for Pap smear. |  |  |  |  |  |
| 3 | If I go to the doctor for Pap smear, my husband's sister or my husband's mother will complain. |  |  |  |  |  |
| 4 | My wife does not want to spend money on Pap smear. |  |  |  |  |  |
| 5 | My sister or my mother believe that I should not spend money on Pap smear |  |  |  |  |  |
| 6 | my husband's sister or my husband's mother believe that I should not spend money on Pap smears |  |  |  |  |  |
| 7 | My wife believes that there is no need for Pap smears until I have a particular problem |  |  |  |  |  |
| 8 | My sister or my mother believe that I don't need a Pap smear until I have a particular problem. |  |  |  |  |  |
| 9 | My husband's sister or my husband's mother believe that I don't need a Pap smear until I have a problem |  |  |  |  |  |
| 10 | My husband advises me to do a Pap smear test. |  |  |  |  |  |
| 11 | My doctor and healthcare providers recommend me that I do a Pap smear test |  |  |  |  |  |
| 12 | My sister or my mother advise me to do a Pap smear test. |  |  |  |  |  |
| 13 | My husband's sister or my husband's mother advises me to do a Pap smear test |  |  |  |  |  |
| 14 | My sister or my mother regularly does a Pap smear |  |  |  |  |  |
| 15 | My husband's sister or my husband's mother regularly does a Pap smear |  |  |  |  |  |
| 16 | If it turns out I have cervical cancer my husband may divorce me |  |  |  |  |  |

Enablers

|  | Please comment on the following questions | I agree very much | I agree | No idea | I disagree | I disagree very much |
| --- | --- | --- | --- | --- | --- | --- |
| 1 | It is costly for me to do a Pap smear test |  |  |  |  |  |
| 2 | I don’t have any training about the necessity of doing of Pap smears yet |  |  |  |  |  |
| 3 | I don't know when to do a Pap smear. |  |  |  |  |  |
| 4 | I don't have access to lab facilities to do a Pap smear test |  |  |  |  |  |
| 5 | Being aware of the benefits of Pap smears has made me think of Pap smears |  |  |  |  |  |
| 6 | I don't have enough time to do a Pap smear test |  |  |  |  |  |

Behavioral intention

|  | Please comment on the following questions | I agree very much | I agree | No idea | I disagree | I disagree very much |
| --- | --- | --- | --- | --- | --- | --- |
| 1 | I have planned to do a Pap smear test on a regular basis (once a year to 3 years and if there is no problem ,every 3 years). |  |  |  |  |  |
| 2 | I intend to use proper nutrition containing vitamin A, vitamin C and folic acid to reduce the risk of cervical cancer |  |  |  |  |  |
| 3 | I want to maintain genital health to reduce the risk of cervical cancer |  |  |  |  |  |
| 4 | I intend to see a doctor as soon as possible if symptoms of danger(such as: Bleeding and abnormal vaginal discharge) occur |  |  |  |  |  |
| 5 | I intend to prevent more than 4 pregnancies |  |  |  |  |  |

Behavior

|  | Please answer on the following questions | Yes | No |
| --- | --- | --- | --- |
| 1 | I do Pap smear test on a regular basis(once a year to 3 years and if there is no problem ,every 3 years). |  |  |
